# Lead-Free BNT–BT_0.08_/CoFe_2_O_4_ Core–Shell Nanostructures with Potential Multifunctional Applications

**DOI:** 10.3390/nano10040672

**Published:** 2020-04-03

**Authors:** Marin Cernea, Roxana Radu, Harvey Amorín, Simona Gabriela Greculeasa, Bogdan Stefan Vasile, Vasile Adrian Surdu, Paul Ganea, Roxana Trusca, Marwa Hattab, Carmen Galassi

**Affiliations:** 1 National Institute of Materials Physics, Atomistilor 405A, 077125 Magurele, Romania; mcernea@infim.ro (M.C.); simona.greculeasa@infim.ro (S.G.G.); paul.ganea@infim.ro (P.G.); 2 Instituto de Ciencia de Materiales de Madrid, CSIC, Cantoblanco, 28049 Madrid, Spain; hamorin@icmm.csic.es; 3 Department of Science and Engineering of Oxide Materials and Nanomaterials, Faculty of Applied Chemistry and Materials Science, University Politehnica of Bucharest, 060042 Bucharest, Romania; bogdan.vasile@upb.ro (B.S.V.); adrian.surdu@upb.ro (V.A.S.); truscaroxana@yahoo.com (R.T.); 4 Research Laboratory of Environmental Science and Technologies, Carthage University, BP.1003, Hammam-Lif, Ben Arous 2050, Tunisia; marouahattab@gmail.com; 5 Faculty of Mathematical, Physical and Natural Sciences of Tunis, University of Tunis Elmanar, Belvedere, Tunis 1002, Tunisia; 6 National Research Council of Italy–Institute of Science and Technology for Ceramics (CNR–ISTEC), Via Granarolo 64, I–48018 Faenza, Italy; carmen.galassi@istec.cnr.it

**Keywords:** oxide materials, sol-gel processes, piezoelectric/ferromagnetic composites, composite core–shell, dielectric properties, magnetic properties, magnetoelectric properties

## Abstract

Herein we report on novel multiferroic core–shell nanostructures of cobalt ferrite (CoFe_2_O_4_)–bismuth, sodium titanate doped with barium titanate (BNT–BT_0.08_), prepared by a two–step wet chemical procedure, using the sol–gel technique. The fraction of CoFe_2_O_4_ was varied from 1:0.5 to 1:1.5 = BNT–BT_0.08_/CoFe_2_O_4_ (molar ratio). X–ray diffraction confirmed the presence of both the spinel CoFe_2_O_4_ and the perovskite Bi_0.5_Na_0.5_TiO_3_ phases. Scanning electron microscopy analysis indicated that the diameter of the core–shell nanoparticles was between 15 and 40 nm. Transmission electron microscopy data showed two–phase composite nanostructures consisting of a BNT–BT_0.08_ core surrounded by a CoFe_2_O_4_ shell with an average thickness of 4–7 nm. Cole-Cole plots reveal the presence of grains and grain boundary effects in the BNT–BT_0.08_/CoFe_2_O_4_ composite. Moreover, the values of the *dc* conductivity were found to increase with the amount of CoFe_2_O_4_ semiconductive phase. Both X-ray photoelectron spectroscopy (XPS) and Mössbauer measurements have shown no change in the valence of the Fe^3+^, Co^2+^, Bi^3+^ and Ti^4+^ cations. This study provides a detailed insight into the magnetoelectric coupling of the multiferroic BNT–BT_0.08_/CoFe_2_O_4_ core–shell composite potentially suitable for magnetoelectric applications.

## 1. Introduction

Multifunctional materials combine materials with different physical properties inti one single structure in order to improve their final functionality [1,2,3]. In particular, multifunctional materials combining both electric and magnetic properties produce a single device component that performs more than one task. These materials show a magnetoelectric effect, namely, the induction of polarization by means of a magnetic field and the induction of magnetization by means of an electric field. In the magnetostrictive–piezoelectric composites, a magnetostrictive phase is mechanically coupled to a piezoelectric phase by an elastic interaction between the two phases. The magnetoelectric (ME) materials can be used for magnetic sensors, microelectromechanical systems (MEMS), and energy harvesters [4,5,6]. Core-shell grain structure is one of the possible ways to combine two properties into one nanostructure and thus achieve advanced performance and multifunctionality due to the availability of a large interfacial area for possible coupling between ferromagnetic and ferroelectric properties [7,8]. Oliveira, P.N. et al. [8], showed that CoFe_2_O_4_:BaTiO_3_ core-shell composite nanoparticles are suitable for biological and biomedical applications. In this paper, we report on the multiferroic core-shell nanostructures of (Bi_0.5_Na_0.5_)_0.92_Ba_0.08_TiO_3_ abbreviated as BNT–BT_0.08_ and CoFe_2_O_4_ for multi-functional devices. BNT–BT_0.08_ and CoFe_2_O_4_ species can form composites that should combine their piezoelectric and magnetic properties and lead to performance surpassing their individual components. These nanocomposites, with a lead-free piezoelectric BNT–BT_0.08_–core and ferrite CoFe_2_O_4_–shell, have not been well studied to date [9]. We focused on CoFe_2_O_4_/BNT–BT_0.08_ composites with core–shell architectures and various molar ratios since there is only one previously published report on 0.65BaTiO_3_–0.35Bi_0.5_Na_0.5_TiO_3_/CoFe_2_O_4_ composites with 0–3 connectivity [10]. There are also few reports on hybrid piezoelectric/ferromagnetic structures, such as: CoFe_2_O_4_/BaTiO_3_ [8,11,12,13,14,15,16], Pb(Zr_0.52_Ti_0.48_)O_3_/NiFe_2_O_4_ [12] and NiFe_2_O_4_/BaTiO_3_ [17] core–shell composites in which the authors studied the influence of the variation of the ferrite fraction on the magnetic properties of the multiferroic core–shell–type nanostructures.

The aim of this work is to prepare and investigate the influence of CoFe_2_O_4_ concentration on the dielectric, piezoelectric, magnetic and magnetoelectric properties of the nanoparticles in a core-shell piezoelectric–magnetic multiferroic structure, with the molar ratio BNT–BT_0.08_/CoFe_2_O_4_= 1:0.5, 1:1 and 1:1.5.

## 2. Experimental Section

### 2.1. Materials and Methods

In order to fabricate BNT–BT_0.08_/CoFe_2_O_4_ core–shell materials, in the first step, (Bi_0.5_Na_0.5_)_0.92_Ba_0.08_TiO_3_, abbreviated as BNT–BT_0.08_, powder was prepared by a simple sol-gel method. Bismuth (III) acetate ((CH_3_COO)_3_Bi, 99.99%), sodium acetate (CH_3_COONa, 99.995%), barium acetate ((CH_3_COO)_2_Ba, 99%), titanium (IV) isopropoxide (Ti[OCH(CH_3_)_2_]_4_) in isopropanol, acetic acid, acetylacetone and formamide were used as starting materials. All reagents were provided by Sigma–Aldrich, St. Louis, MO, USA. The acetate salts were separately dissolved in acetic acid, at 100 °C (sodium and barium acetates) and 200 °C (bismuth acetate). BaTiO_3_ precursor sol was prepared by adding the acetic solution of Ba^2+^ to the titanium (IV) isopropoxide in isopropanol; the as–obtained sol was stabilized with acetylacetone. The solution of Bi^3+^ was added to the acetic Na^+^ solution and a clear mixture was obtained. The amount of titanium (IV) isopropoxide in isopropanol required to obtain Bi_0.5_Na_0.5_TiO_3_, was mixed with acetylacetone and then added to the solution of Bi^3+^ and Na^+^. This solution was added to the BaTiO_3_ precursor sol and the BNT–BT_0.08_ precursor sol was obtained. This last sol was homogenized by magnetic stirring at 75 °C for 3h and transformed into gel at 90 °C. The dried gel was calcined at 700 °C for 3 h and the BNT–BT_0.08_ powder was obtained. The CoFe_2_O_4_ sol precursor was prepared by a sol–gel–combustion route, from cobalt(II) acetate tetrahydrate (Co(CH_3_CO_2_)_2_·4H_2_O, 99.995%, Sigma–Aldrich), iron(III) nitrate nonahydrate (Fe(NO_3_)_3_·9H_2_O, 99.99%, Sigma–Aldrich), citric acid (C_6_H_8_O_7_, 99%), ethanol, acetic acid and distilled water. Ethanol, acetic acid and distilled water were used as solvents. Citric acid was used as the chelating agent and fuel. Practically, iron(III) nitrate acts as an oxidizing agent and citric acid acts as a reducing agent with an exothermic reaction. The maximum heat was released when the oxidizer to organic fuel molar ratio (in this case citrate/nitrate ratio, CA/NO^3–^) became equal to unity [18]. When excessive HNO_3_ was added, the combustion became more violent due to the displacement of the chemical balance in the sense of the oxidant (NO^3–^) which is in excess [19]. The auto–combustion of citrate–nitrate gel method has been patented by Chakrabarti et al. [20], in order to synthesize multicomponent oxides. This method was based on gelling of a nitrate solution of the desired metals together with some organic fuel (glycine, citric acid, sucrose, urea, or other water soluble carbohydrates) followed by combustion due to the exothermal redox reaction between nitrate ions and the fuel. This process has the advantage of rapidly producing fine and homogeneous powder. To prepare CoFe_2_O_4_ precursor sol, we used the following strategy: the iron nitrate and citric acid were dissolved in ethanol separately, at room temperature. The cobalt acetate was dissolved in ethanol, acetic acid and water. A solution of iron nitrate was added to cobalt acetate solution, and then the citric acid solution was added to the mixture solutions of Fe and Co, to form the cobalt ferrite precursor sol. The citrate/nitrate molar ratio, CA/NO^3-^ was 1:1. The as-obtained sol was used to prepare BNT–BT_0.08_/CoFe_2_O_4_ core–shell composites with various molar ratios BNT–BT_0.08_/CoFe_2_O_4_ = 1:0.5 (sample S_1_; 35.71 wt% CoFe_2_O_4_ and 64.49% BNT–BT_0.08_), 1:1 (sample *S*_2_; 52.41 wt% CoFe_2_O_4_ and 47.59% BNT–BT_0.08_), and 1:1.5 (sample *S*_3_; 68.77 wt% CoFe_2_O_4_ and 31.23% BNT–BT_0.08_). For this purpose, the BNT–BT_0.08_ powder was added to the precursor sol of the CoFe_2_O_4_ and kept in suspension by continuous stirring at 80 °C until the BNT–BT_0.08_/CoFe_2_O_4_ core–shell powders were formed. The as–obtained powders were calcined at 700 °C for 1 h in air. The pellets of BNT–BT_0.08_/CoFe_2_O_4_ obtained by uniaxial pressing were sintered at 1100 °C for 15 min in air.

### 2.2. Characterization of the Core–Shell Materials

The crystallographic structure and composition of the core–shell composites were examined using a PANalytical Empyrean diffractometer (PANalytical, Almelo, The Netherlands ). CuK*_α_* radiation (wavelength 1.5418 Å), two bounce Ge 220 monochromator (PANalytical, Almelo, The Netherlands) and Bragg–Brentano diffraction geometry were employed. XRD data were acquired at 25 °C with a step–scan interval of 0.02° and a step time of 10 s, in the range of 20–80°. The phase content was estimated by Rietveld refinement, using HighScore Plus 3.0e software (PANalytical, Almelo, The Netherlands). The background was corrected with the pseudo-Voigt function. The values of the *R* factors: *R_profile_*, *R*_expected_, weighted *R* profile and *χ*^2^, as the crystallographic parameters and average crystallite size are calculated. The microstructure of the core–shell heterostructures was assessed using a scanning electron microscope (Inspect F50, FEI, Hillsboro, OR, USA) with a field emission gun and a transmission electron microscope (Tecnai^TM^ G2 F30 S-TWIN, FEI, Hillsboro, OR, USA) possessing a line–resolution of 1 Å, in high-resolution transmission electron microscopy (HRTEM) mode. The crystalline structure of the samples was investigated by selected area electron diffraction (SAED). X-ray photoelectron spectroscopy (XPS) measurements were performed using a Kratos Ultra DLD Setup spectrometer (Kratos Analytical Ltd., Manchester, UK). A monochromatized Al K*_α_*_1_ (1486.74 eV) radiation X-ray source was used, operating at 15 kV, an emission current of 15 mA at a pressure in the analysis chamber of 1.2 × 10^−9^ mbar. The dielectric properties of the samples were studied at room temperature in 10^–2^–10^7^ Hz range of frequency, in the metal–dielectric–metal (MFM) configuration, where the electrodes consist of silver paste, using a 4194A Impedance/Gain–Phase Analyzer. A nominal voltage equal to 0.5 mV was applied. The Mössbauer experiments were performed using a constant acceleration spectrometer (triangular wave form) and a ^57^Co (Rh matrix) source. The spectra were acquired at room temperature, in transmission geometry. The NORMOS computer program [21] was used for least-squares fitting of the Mössbauer spectra. The isomer shifts are reported relative to a-Fe α-Fe at room temperature. The magnetic hysteresis loops of the composites were measured at room temperature by a vibrating sample magnetometer (Lake Shore 7404–s VSM, Westerville, OH, USA). Finally, both transverse 3-1 and longitudinal 3-3 magnetoelectric responses were characterized with a homebuilt system using a combination of two Helmholtz coils. The system was designed to independently provide a static magnetic field up to 1 kOe to magnetize the material, and an alternate magnetic field of 10 Oe at 1 kHz that functions as the stimulus, while the magnetoelectric voltage response was monitored with a lock-in amplifier. Previously, the composites were poled at room temperature by applying increasingDCelectric fields while keeping leakage currents below the threshold.

## 3. Results and Discussion

### 3.1. Structure and Microstructure Characterization

**Figure 1:**
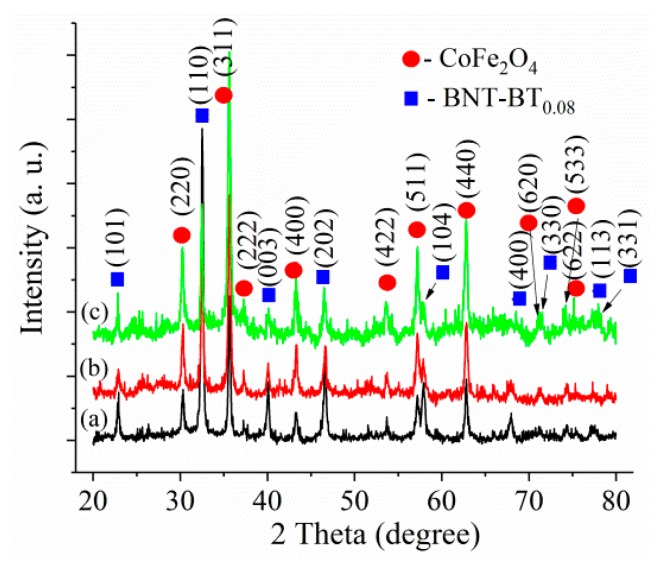
XRD patterns of the BNT–BT_0.08_/CoFe_2_O_4_ core–shell powders: (**a**) sample *S*_1_, (**b**) sample *S*_2_, (**c**) sample *S*_3_ calcined at 700 °C for 1 h in air.

Figure 1 shows the XRD patterns of the BNT–BT_0.08_/CoFe_2_O_4_ core–shell powders calcined at 700 °C for 1h in air.

As can be seen, from Figure 1, the diffraction peaks of the Bi_0.5_Na_0.5_TiO_3_ tetragonal perovskite structure (space group *P4bm*) (JCPDS 011–4032) [22] are higher for the BNT–BT_0.08_/CoFe_2_O_4_ = 1:0.5 and decrease in intensity with the increase of the CoFe_2_O_4_:BNT–BT_0.08_ ratio. At the same time, the peaks of the cubic spinel CoFe_2_O_4_ phase (space group Fd3m) [23] increase with increasing CoFe_2_O_4_. No secondary phases were detected by X–ray diffraction analyses in these nanocomposite samples. Detailed structural information for the BNT–BT_0.08_/CoFe_2_O_4_ core–shell powders was obtained using Rietveld refinement and presented in Table 1. The values of the average crystallite size for CoFe_2_O_4_ phases decrease with an increasing amount of CoFe_2_O_4_. It should be mentioned that the degree of tetragonality of BNT–BT_0.08_, calculated as the ratio of the lattice parameters c and a, decreases as the content of CoFe_2_O_4_ increases. The core-shell samples have similar degree of tetragonality as for BNT–BT_0.08_, considering the same synthesis. This observation could be attributed to the strain induced in the BNT–BT_0.08_ phase during CoFe_2_O_4_ crystallization, surrounding the BNT–BT_0.08_. Furthermore, as indicated in Section 3.2, the dielectric constant decreases with increasing CoFe_2_O_4_ content, thus having a similar behavior pattern as the degree of tetragonality. It is well known that the tetragonal phase of the perovskite materials has an increased dielectric constant compared with the cubic one. 

**Figure 2:**
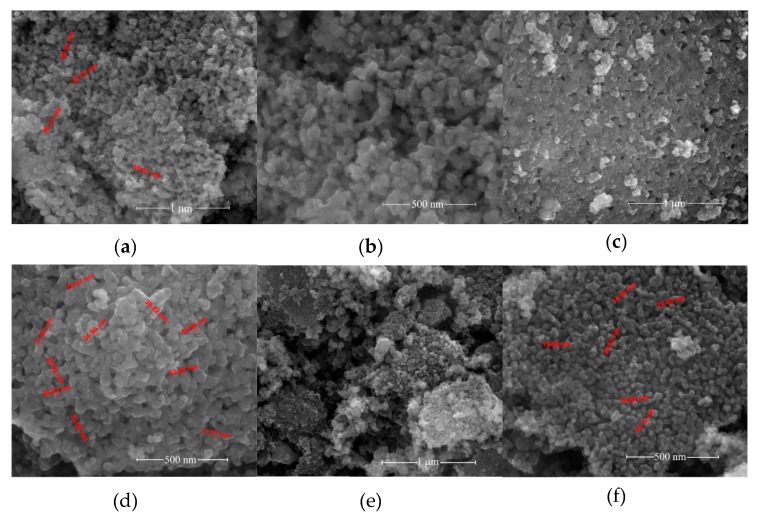
SEM micrographs at two magnifications of the BNT–BT_0.08_/CoFe_2_O_4_ core–shell powders samples: (**a**), (**b**) *S*_1_; (**c**), (**d**) *S*_2_ and (**e**), (**f**) *S*_3_.

Figure 2 shows SEM images of the BNT–BT_0.08_/CoFe_2_O_4_ core–shell powders calcined at 700 °C for 1h in air.

As can be seen in Figure 2, all the powders show core–shell nanoparticles with sizes between 15 and 40 nm. The powders present a uniform morphology; the particles having a polyhedral shape. In addition, the powders have a high degree of agglomeration due to the magnetic cobalt ferrite shell. The crystal structures of the core-shell nanopowders (samples *S*_1_–*S*_3_) have also been examined by TEM.

**Figure 3:**
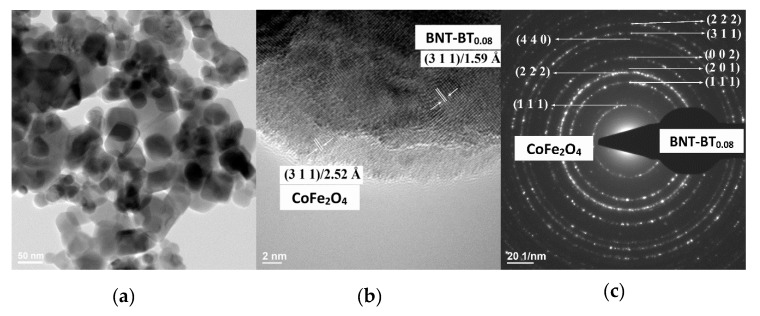
(**a**) Bright field TEM (BF–TEM) image, (**b**) High Resolution-Transmission Electron Microscopy (HR–TEM) and (**c**) corresponding selected area electron diffraction (SAED) patterns of the BNT–BT_0.08_/CoFe_2_O_4_ core–shell powder (sample *S*_1_).

**Figure 4:**
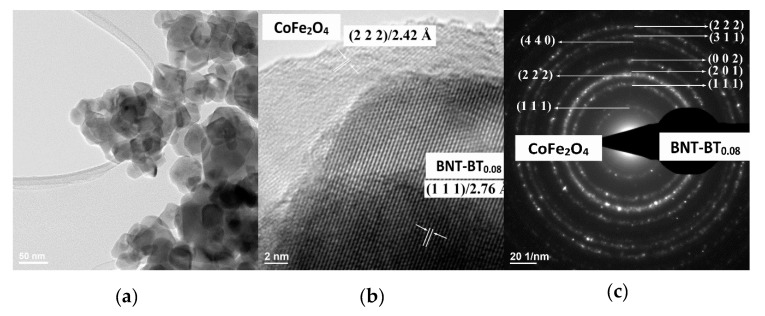
(**a**) BF–TEM image, (**b**) HR–TEM and (**c**) corresponding SAED patterns of the BNT–BT_0.08_/CoFe_2_O_4_ core–shell powder (sample *S*_2_).

**Figure 5:**
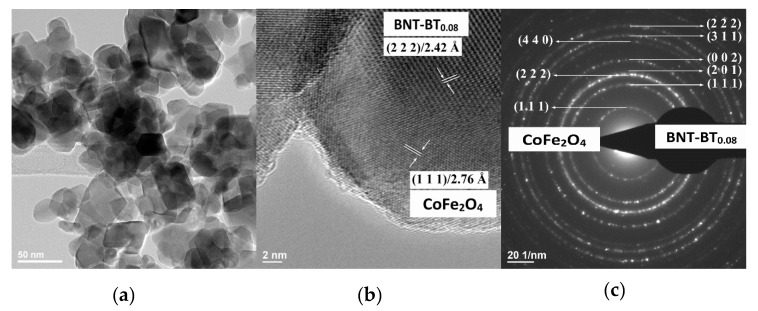
(**a**) BF–TEM image, (**b**) HR–TEM and (**c**) corresponding SAED patterns of the BNT–BT_0.08_/CoFe_2_O_4_ core–shell powder (sample *S*_3_).

The low-resolution TEM images (bright field images (BF–TEM images)) (Figure 3a, Figure 4a and Figure 5a) show nanoparticles, which show an agglomeration trend. The corresponding high–resolution TEM images for the nanoparticles (Figure 3b to Figure 5b) show nanoparticles of the calcined BNT–BT_0.08_/CoFe_2_O_4_ core/shell nanostructures with an estimated shell thickness of 4, 6 and 7 nm, respectively. HR–TEM images of the samples *S*_1_–*S*_3_ reveal respective fringe spacings of 2.42 Å and 2.76 Å, which correspond to the interplanar spacings along the BNT–BT_0.08_ (222) and CoFe_2_O_4_ (111) directions. The SAED patterns present interplanar spacings of (222), (311), (002), (201) and (111) corresponding to tetragonal BNT–BT_0.08_ and (440), (222) and (111) interplanar spacings corresponding to the cubic CoFe_2_O_4_ phases (Figure 3c to Figure 5c). For the samples S_1_–S_3_, the HR–TEM and circular distinct ring patterns (SAED patterns) corresponding to the different lattice planes confirm the core–shell heterostructure of the grains, with BNT–BT_0.08_ and CoFe_2_O_4_ phases. Also, HR–TEM micrographs of core–shell interface clearly show the existence of an interface between grain core and layer shell for the powder samples analyzed.

**Figure 6:**
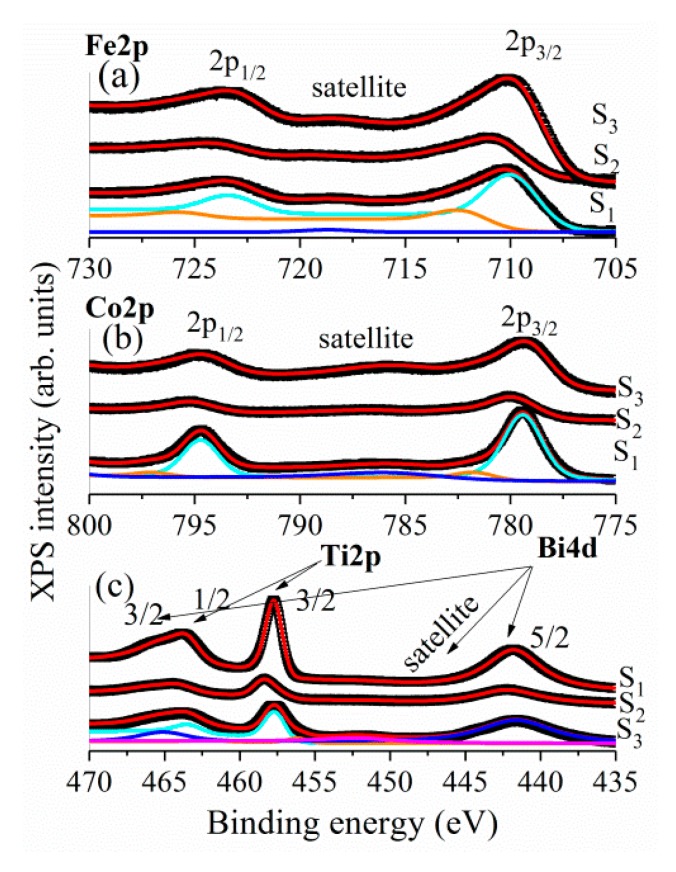
Core levels photoemission of: (**a**) Fe 2p; (**b**) Co 2p; (**c**) Ti 2p and Bi 4d measured on the BNT–BT_0.08_/CoFe_2_O_4_ core–shell powders (samples *S*_1_, *S*_2_ and *S*_3_).

XPS analyses were performed with the purpose of identifying the chemical composition and the valence of the cations, thus determining the influence of the CoFe_2_O_4_ on the magnetic properties of the BNT–BT_0.08_/CoFe_2_O_4_ core–shell composites. To our knowledge, this is the first study using XPS analysis on BNT–BT_0.08_/CoFe_2_O_4_ core–shell composites. The XPS spectra of BNT–BT_0.08_/CoFe_2_O_4_ for all pellets samples are illustrated in Figure 6. The core level spectra of the BNT–BT_0.08_/CoFe_2_O_4_ core–shell composites (Fe 2p, Co 2p, Ti 2p, Bi 4d) were deconvoluted using Voigt functions (Lorentzian and Gaussian widths) having a different inelastic background for each component [24,25,26]. For the analyses of the XPS data, all the binding energies for all peaks were calibrated using the value of the binding energy of C 1s of 284.8 eV. It should be mentioned that the C 1s photoelectron peak in all powders (not shown here) was observed at 283.6 eV, while for samples *S*_1_–*S*_3_ its values were of 283.13 eV, 284.09 eV and 285.09, respectively. 

The Fe 2p main peak was fitted with two doublets with binding energies at 710.5 eV and 712.6 eV attributed to Fe^3+^ ions in octahedral and tetrahedral sites, respectively [27]. It was calculated that around 80% of Fe^3+^ ions are located in octahedral sites while 20% are in tetrahedral sites. The satellite peak of Fe 2p_3/2_, located approximately 8 eV higher than the main peak, is clearly distinguishable. In a similar manner, the Co 2p XPS spectra were fitted with two doublets (Figure 6b) with binding energies at 779.7 eV and 781.4 eV and attributed to Co^2+^ ions in octahedral sites and tetrahedral sites, respectively. Furthermore, the signal at 785.9 eV was assigned to the satellite peak of Co 2p_3/2_ main line.

**Figure 7:**
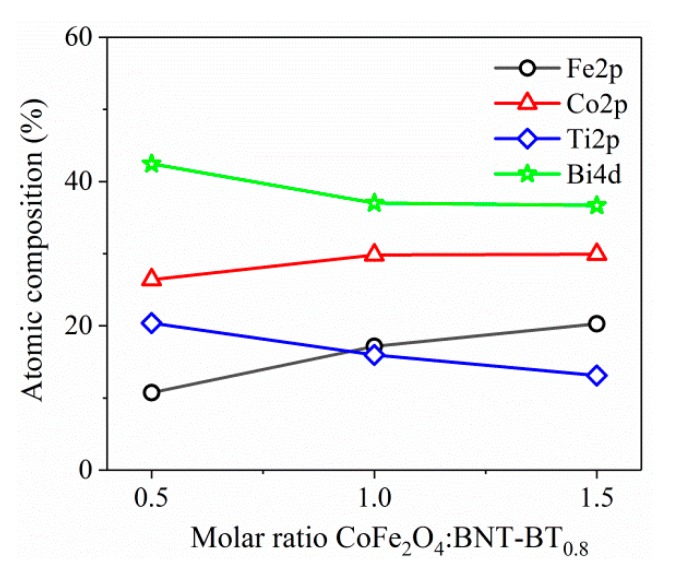
The atomic composition, determined from core levels photoemission of Fe 2p, Co 2p, Ti 2p and Bi 4d, for the samples *S*_1_–*S*_3_.

Figure 6c shows the XPS spectra of the Ti 2p and Bi 4d. One can notice that the peaks of Ti 2p_1/2_ and Bi 4d_3/2_ are partially overlapped, resulting in a broad bump around 464 eV [28]. Thus, the XPS peaks with binding energies at 441.7 eV and 465.1 eV are attributed to Bi 4d_5/2_ and Bi 4d_3/2_, which correspond to Bi^3+^, while the peaks at 457.7 and 463.6 eV are attributed to Ti 2p_3/2_ and Ti 2p_1/2_, corresponding to Ti^4+^. The atomic composition was calculated, for all the investigate samples, by taking into account the integral areas provided by the deconvolution procedure, normalized to the XPS atomic sensitivity factors described in [29] (Figure 7). As expected, by increasing the molar ratio BNT–BT_0.08_/CoFe_2_O_4_ from 1:0.5 to 1:1.5 the composition of Fe^3+^ and Co^2+^ ions increases.

### 3.2. Complex Impedance Spectrum Analysis

**Figure 8:**
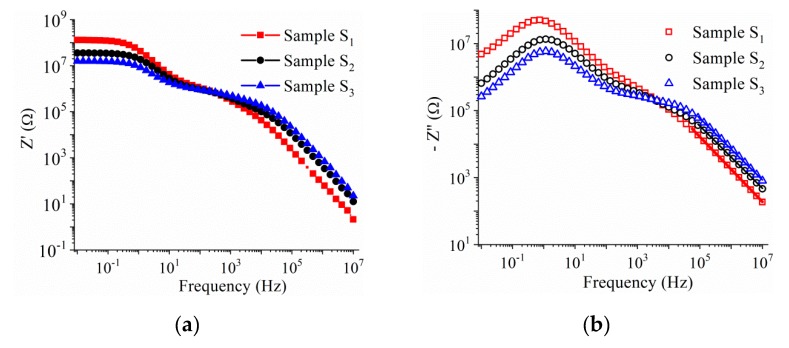
Frequency dependence of the (**a**) real and (**b**) imaginary part of impedance measured at room temperature. The dots represent the experimental data whilst the solid lines represent the best linear fit.

The contribution from the sample-electrode interface, grain (intrinsic), grain boundary (extrinsic) and bulk resistance and capacitance is reflected in the charge transport mechanism and the macroscopic dielectric constant in polycrystalline materials. Using complex impedance spectroscopy measurements one can gain essential information regarding their individual contribution by analyzing the response of a crystalline material to an alternating field [30,31,32]. The electric properties of a material such as its resistive and capacitive parts are represented in terms of complex dielectric permittivity, *ε*, complex impedance, *Z*, and electric modulus, *M*, which are related to each other as: *Z* = *Z*′ + *iZ*″; *M* = 1/*ε* = *M*′ + *iM*″, where (*Z*′, *M*′) and (*Z*″, *M*″) are the real and imaginary components of impedance and modulus, respectively. Figure 8a,b show the variation of the real part (*Z*′) and the imaginary part (*Z*″) of the impedance with frequency, for the BNT–BT_0.08_/CoFe_2_O_4_ ceramics samples *S*_1_–*S*_3_, measured at room temperature, in the range 10^−2^–10^7^ Hz. One can observe that both values of the real and imaginary part of the impedance decrease with the rise of CoFe_2_O_4_ concentration in the composite, up to a frequency of 10 kHz, while at high frequencies the trend is reversed. It was found that for all the samples (*S*_1_–*S*_3_), the loss spectrum, *Z*″ vs. frequency, shows the appearance of peaks with maxima at 1 Hz, indicating dielectric relaxation processes in this region. Moreover, in the high-frequency region (10^5^–10^7^ Hz), the loss spectrum appears to have a linear decrease with a slope about −1, which can be related to the exponent used in cases of polycrystalline materials to explain the Constant–Phase–Element (CPE) behavior [33,34].

**Figure 9:**
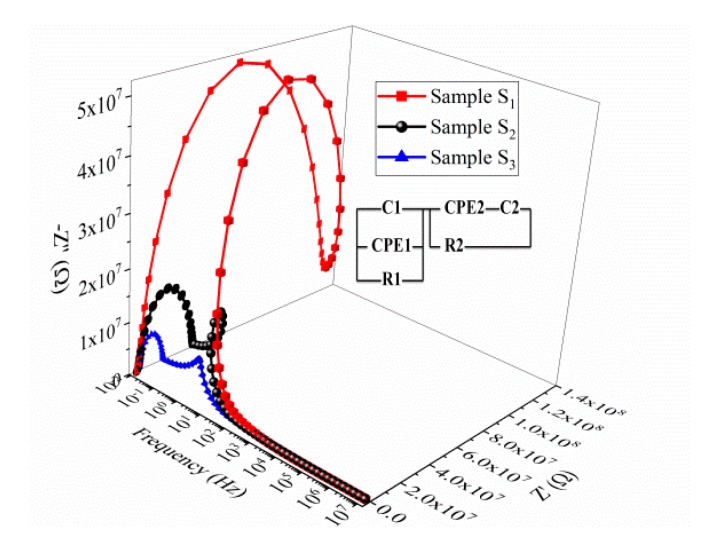
3D Cole–Cole plots of impedance, measured at room temperature, of the BNT–BT_0.08_/CoFe_2_O_4_ sintered ceramics. The symbols represent the experimental data whereas the solid lines are the results of the fitting procedure using the equivalent circuit modeled with the EIS Spectrum Analyzer.

Figure 9 shows the Cole–Cole plots of impedance for the BNT–BT_0.08_/CoFe_2_O_4_ ceramics for samples *S*_1_–*S*_3_. In impedance plots (Cole-Cole plots), the radii of semicircles are equivalent to the resistive behavior of ceramic samples. With the increase of the CoFe_2_O_4_ concentration the radii of Cole–Cole plots start decreasing, indicating a decrease in resistivity (i.e., an increase of the conductivity with the CoFe_2_O_4_ amount, see Table 2).

The two semicircles present in the complex plots are a signature of the corresponding electrical process taking place in the material. Generally, the semicircles with smaller radii (i.e., lower resistance) will appear at higher frequencies, whereas at lower frequencies semicircles with higher radii will appear. In a polycrystalline material, in the high frequency region, the interface states of the grain boundaries cannot follow the ac signal. Thus, the semicircles result from the contribution of crystalline grains, while at low frequencies the semicircles correspond to the contribution of the grain boundaries [35,36]. The complex impedance data has been interpreted by using an appropriate equivalent circuit, which is a series arrangement of a resistance and capacitance that are connected in parallel, attributed to the grain contribution denoted C1, R1 and CPE1. The second component contains a parallel couple of a resistance and capacitance attributed to the grain boundary, denoted C2, R2 and CPE2. The parameters for the grain and grain boundary were estimated by fitting experimental data, modeled with the EIS Spectrum Analyzer, with an appropriate equivalent circuit (inset of Figure 9). 

**Figure 10:**
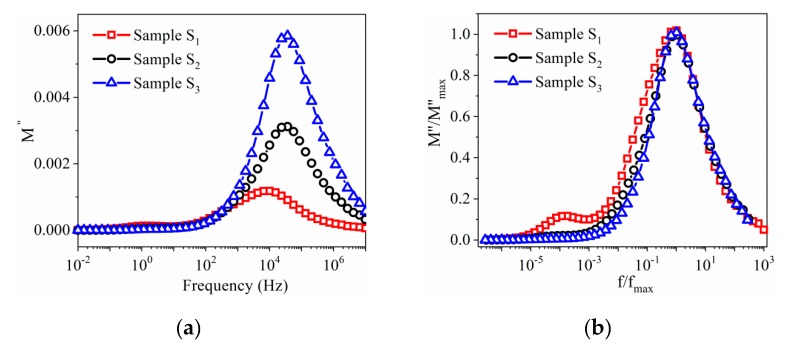
(**a**) Frequency dependence of imaginary part (*M*″) of the dielectric modulus; (**b**) the normalized plots (*M*″/*M*″ max) versus log (*f*/*f*_max_).

The frequency dependence of the imaginary part of the electric modulus (*M*″), shown in Figure 10, exhibits a very clear relaxation peak whose maximum position (around 10^4^ Hz) shifts toward higher frequencies as the CoFe_2_O_4_ content in the composite increases. Earlier studies [18] showed the appearance of the relaxation in the same range of frequencies. Figure 10b shows the normalized plot (*M*″/*M*_max_″) versus log (*f*/*f*_max_) of the modulus for the three samples *S*_1_–*S*_3_. It is evident that the measured curves do not completely overlap. In addition, with decreasing of the CoFe_2_O_4_ concentration in the composite, the main peak gets broader, and a second peak appears (Figure 10b). The presence of a second peak in the *M*″ spectra, at 1 Hz, with decreasing the CoFe_2_O_4_ content, might be a signature of the suppression of some dynamical processes or the overlapping of either two effects (contribution of the grain and grain boundaries). This behavior is very clearly seen in the Cole–Cole plots of the impedance spectra, where, at high frequencies, a second depressed semicircle is more easily observed in the case of the BNT–BT_0.08_/CoFe_2_O_4_ core–shell composite with a molar ratio of 1:0.5.

**Figure 11:**
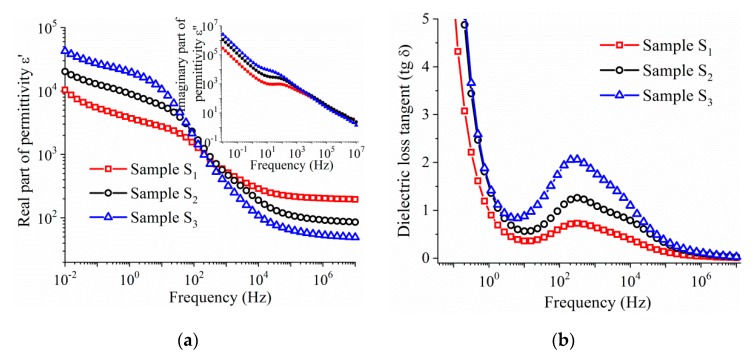

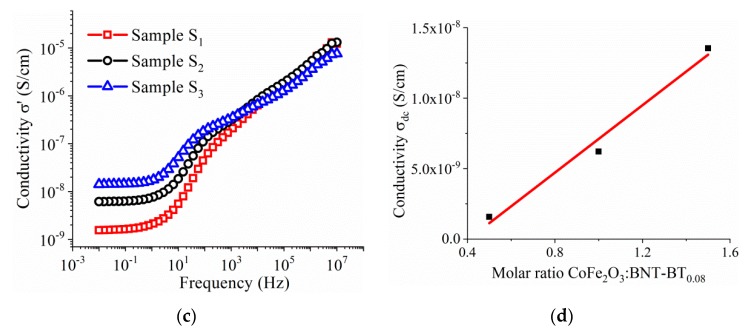
Frequency dependence of: (**a**) the real part of the permittivity (*ε*′). The inset shows the imaginary part of the permittivity, (**b**) loss tangent, (**c**) AC conductivity for the sintered ceramics samples *S*_1_–*S*_3_, and (**d**) the DC conductivity as function of the CoFe_2_O_4_ content.

Figure 11a shows the frequency dependence of the real part of relative permittivity (*ε*′) of the core–shell BNT–BT_0.08_/CoFe_2_O_4_ pellets sintered at 1100 °C for 10 min in air. With increasing frequency, *ε*′ exhibits high values in the low-frequency range and decreases with further increase of frequency. The dielectric constant decreases with increasing amounts of CoFe_2_O_4_ and decreases in resistive BNT–BT_0.08_ phases, up a frequency of 10 kHz, while at high frequencies the trend is reversed. The relaxation frequency that characterizes the dispersion in capacitance can be obtained from the maximum in the corresponding dielectric loss spectrum. The frequency dependence of the *ε*″ (illustrated in the inset of Figure 11a) shows a relaxation peak whose amplitude is shifted towards the high frequency side with decreasing the CoFe_2_O_4_ content. 

The change of the total conductivity with the rise in frequency is shown in Figure 11c, where the low-frequency region (the plateau) is dominated by *dc* conduction. The value of the frequency-independent part of conductivity (*σ*_dc_) was evaluated from the *ac* conductivity by fitting the experimental data in the low frequency range using Joncher’s power law (Figure 11c) *σ*_ac_ = *σ*_dc_ + *Aω^n^*, where *A* is constant, *ω* = 2π*f* is the angular frequency of the applied electric field and n is a power law exponent having a value lying between 0 and 1 [37,38]. It is observed that the values of the *dc* conductivity increase linearly with the amount of semi-conductive CoFe_2_O_4_ phase in the pellets (Figure 11d). Moreover, beyond 1 kHz, the values of the conductivity appear to merge at higher frequencies.

### 3.3. Mössbauer Spectroscopy Results

**Figure 12:**
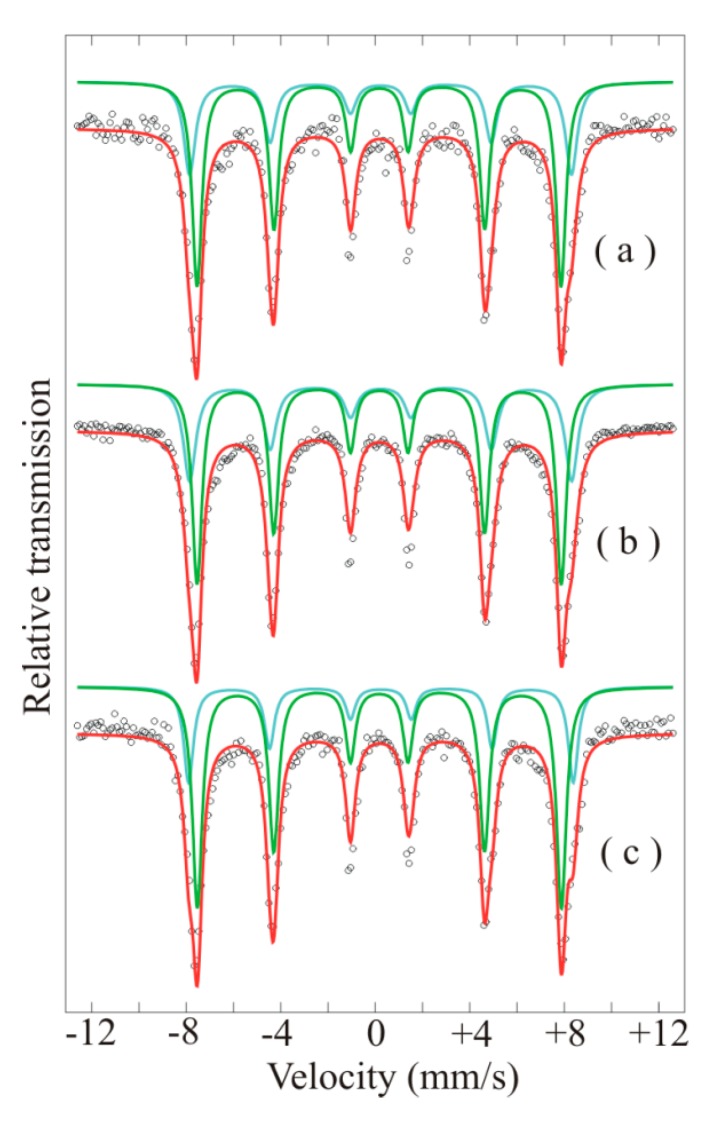
Mössbauer spectra of the powder samples *S*_1_ (**a**), *S*_2_ (**b**) and *S*_3_ (**c**).

Co ferrite is an inverse spinel where, ideally, the tetrahedral sites are occupied by half of the Fe^3+^ ions, while the other half of the Fe^3+^ ions and the Co^2+^ ions are located on the octahedral sites. In real samples, cation disorder is common and the inversion degree is only partial. Some of the Co^2+^ ions are positioned on tetrahedral sites as well. Cationic distribution plays a crucial role in magnetism. However, these correlations are rarely shown in the literature. In this respect, ^57^Fe Mössbauer spectroscopy is a powerful method to probe the nature of magnetic ordering of Fe in ferrites, especially when partial inversion is present. The Mössbauer spectra of the powder samples are shown in Figure 12. Mössbauer spectra of all samples exhibit magnetically split sextets typical to ferrimagnetic spinels. The spectra were fitted using two magnetic sublattices corresponding to the Fe^3+^ ions located on the tetrahedral (*T*) and octahedral (*O*) coordination, respectively, coordinated by four and six oxygen atoms. No doublet due to superparamagnetic relaxation states or paramagnetic phases was observed. The hyperfine parameters and the relative areas of the two components, as well as the inversion degree and formula unit for the CoFe_2_O_4_, are shown in Table 3. Typical isomer shift (IS) and hyperfine magnetic field (Bhf) values for Co ferrites were obtained [39]. Quadrupole splitting values were very small (~10^−2^–10^−3^ mm/s) and are rather considered quadrupole corrections.

The chemical formula unit of the partially inverted CoFe_2_O_4_, giving the cationic distribution in the tetrahedral and octahedral positions, can be derived from the inversion parameter *γ*. The inversion degree is defined as the fraction of tetrahedral sites occupied by Fe^3+^ ions:

(1) $${\left(Co_{1-\gamma }Fe\gamma \right)}^{Tetr}{\left[Co_{\gamma }Fe_{2-\gamma }\right]}^{Oct}O_{4}$$

CoFe_2_O_4_ is completely inversed when *x* = 1. The degree of inversion can be estimated from the intensity ratio of the tetrahedral and octahedral sites obtained from the Mössbauer spectra, *I_T_/I_O_*, by the following relation:

(2) $$\frac{I_{T}}{I_{0}}=\frac{f_{T}}{f_{0}}\frac{\gamma }{2-\gamma }$$

where *f_T_*/*f_O_* is the ratio of the recoilless fractions and is assumed to be 0.94 at room temperature and 1 at low temperatures [40].

Using Equation (2), the inversion degree and cationic distribution of the CoFe_2_O_4_ were obtained and are shown in Table 3, showing a partial inversion of CoFe_2_O_4_. The Mossbauer parameters account for Co ferrite in all three samples and are very similar, in agreement with [39]. The relative area of the sublattices are considerably influenced by cation occupation.

### 3.4. Magnetic Characterization

**Figure 13:**
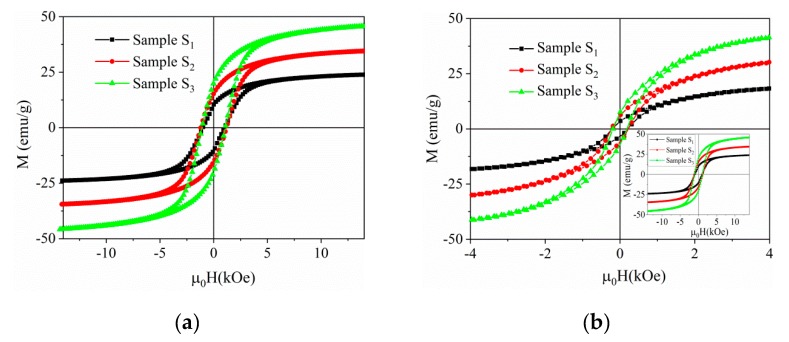
Room temperature magnetization hysteresis loops of BNT–BT_0.08_/CoFe_2_O_4_ core–shell powders (**a**) and ceramics (**b**) in 1:0.5, 1:1 and 1:1.5 molar ratios (sample *S*_1_, sample *S*_2_ and sample *S*_3_). The inset of (**b**) shows an extended view of the corresponding hysteresis curves.

Figure 13 shows the magnetic field dependences of the magnetization measured at room temperature by vibrating sample magnetometry for BNT–BT_0.08_/CoFe_2_O_4_ core–shell nanocomposites, powders and ceramics. The extracted magnetic parameters are listed in Table 4. All samples showed ferrimagnetic-like behavior at room temperature. At the maximum applied field of 15 kOe, the magnetization of the samples was not completely saturated. Therefore, the law of approach to saturation (LAS) was applied:

(3) $$M=M_{s}\left(1-\frac{b}{H^{2}}\right)+kH$$

where *M* is the magnetization, *H* is the applied magnetic field, *M_S_* is the saturation magnetization and *κH* is the so-called forced magnetization. The forced magnetization term can be neglected at room temperature in relatively low fields.

**Figure 14:**
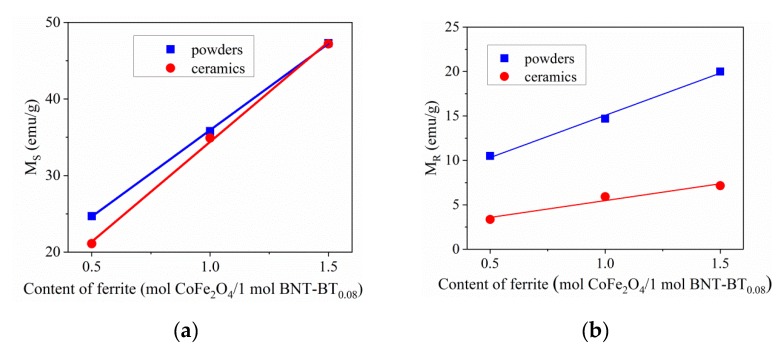
Room temperature saturation (**a**) and remanent (**b**) magnetization values of BNT–BT_0.08_/CoFe_2_O_4_ core–shell powders and ceramics vs. content of CoFe_2_O_4_ (the lines connecting the experimental data are linear fittings).

The saturation and remnant magnetization values of the BNT–BT_0.08_/CoFe_2_O_4_ core–shell nanostructures as a function of CoFe_2_O_4_ concentration are shown in Figure 14a,b.

As expected, the saturation and remanent magnetization values of the core–shell BNT–BT_0.08_/CoFe_2_O_4_ powders and ceramics increase linearly with the content of the magnetic CoFe_2_O_4_ coating while the amount of non-magnetic BNT–BT_0.08_ remains constant in the three samples. Moreover, the *M*_S_ values were roughly similar for the powders and ceramics composites, suggesting that the sintering treatment does not strongly influence the saturation, given a certain concentration. However, the increase is not consistent with the ferrite content evolution due to different cationic distributions as evidenced by Mössbauer spectroscopy. Coercivity values in powder samples are high—even higher than single phase CoFe_2_O_4_ nanoparticles [32]—making the powder samples suitable for hard magnetic applications. The ceramics samples show thin magnetic hysteresis loops illustrating a soft magnetic behavior (Figure 13b). The coercivity is reduced multiple times in the ceramic samples relative to their powder counterparts (see Table 4). For both powder and ceramic samples, coercivity presents a non-monotonous variation with CoFe_2_O_4_ content: H_C_ values for sample *S*_3_ (1.5 mol CoFe_2_O_4_/1 mol BNT–BT_0.08_) are higher than *S*_1_ (0.5 mol CoFe_2_O_4_/1 mol BNT–BT_0.08_) but lower than *S*_2_ (1 mol CoFe_2_O_4_/1 mol BNT–BT_0.08_). The lower coercivity of *S*_3_ may be related to a decrease of the stress anisotropy (due to microstructural strain) or to a decrease of the density of structural defects, similar to [42]. Further, the hysteresis loops of the powder samples are more closely approaching a rectangular shape relative to the corresponding ceramics, inducing higher squareness ratio values. Contrary to the ceramic samples, the hysteresis loops of the powder samples show a squareness ratio close to 0.5, thus approaching the ideal Stoner-Wohlfart case [43] for non-interacting uniaxial single domain particles with the easy axis being randomly oriented. Relative to other BNT–BT_0.65_/CoFe_2_O_4_ or BaTiO_3_/CoFe_2_O_4_ composites of similar CoFe_2_O_4_ content reported in the literature, our powder sample results show considerably higher saturation magnetization and coercivity values (see Table 4). The high-saturation magnetization obtained in Ref. [30] was achieved for a very high amount of CoFe_2_O_4_ relative to the BNT-BT_0.08_ component, approaching the case of the single phase CoFe_2_O_4_ nanoparticles [32]. The saturation magnetization obtained from magnetic measurements is reduced relative to the corresponding values calculated from the Mössbauer spectroscopy results. These differences cannot be explained solely by a partially inverted structure within the Néel model of ferrimagnetism of collinear spins. Spin canting phenomena can occur as a result of alteration of cation distribution due to chemical disorder (affecting the super-exchange interactions) and from broken exchange bonds causing magnetic topological frustration at the core-shell interface. The net magnetic moment per formula unit, n_B_ (expressed in μ_B_ units), can be obtained by the following relation:

(4) $$n_{B}=M_{0}\cos\left(\theta_{RCS}\right)-M_{T}$$

where *M*_0_, *M_T_* are the net magnetic moments on the octahedral and tetrahedral sites, and *θ*_RCS_ is the canting angle at the octahedral site.

Using the values of the inversion degree determined by Mössbauer spectroscopy, the resulting canting angle values are approximately 31, 28 and 23 degrees for samples 1, 2 and 3, respectively. It may be observed that higher CoFe_2_O_4_ content induces a smaller canting angle and, therefore, better approaches the collinear spin structure.

### 3.5. Magnetoelectric Voltage Response

**Figure 15:**
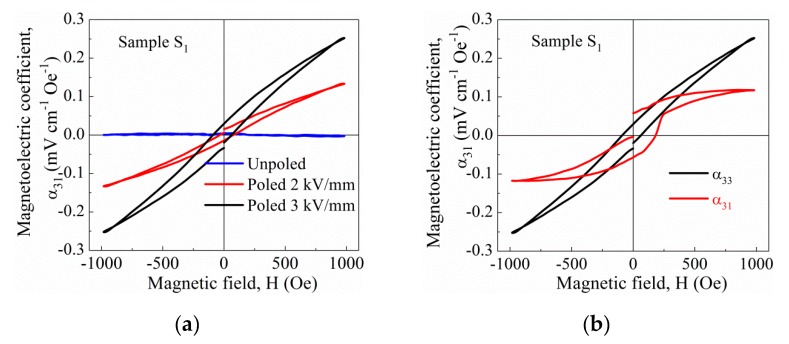
Room-temperature magnetoelectric coefficients as a function of DC magnetic field of BNT–BT_0.08_/CoFe_2_O_4_ core-shell ceramic with a 1:0.5 molar ratio (sample *S*_1_): (**a**) measured in the longitudinal mode (*α*_33_) after poling at increasing electric fields, and (**b**) measured in both longitudinal (*α*_33_) and transverse (*α*_31_) modes after poling at 3 kV/mm.

Figure 15 shows the magnetoelectric voltage coefficients versus the DC magnetic field at room temperature for the BNT–BT_0.08_/CoFe_2_O_4_ core–shell ceramic with a 1:0.5 molar ratio (sample *S*_1_), measured in both longitudinal (*α*_33_) and transverse (*α*_31_) modes.

Sample *S*_1_ was previously poled at 2 and 3 kV/mm for 20 min at room temperature, which resulted in Berlincourt *d*_33_ piezoelectric coefficients of 2 and 5 pC/N. Hot poling was not possible because of non-negligible conduction, and higher electric fields resulted in large leakage currents that prevent the poling process. The *d*_33_ values achieved are rather low compared to figures from other particulate composites [3,10]. However, two issues must be taken into account: the high amount of CoFe_2_O_4_ phase in the composites, and the type of core-shell connectivity of these samples, in which the shell corresponds to the magnetic phase. This results in high conductivity values that prevent the application of high electric fields. Nevertheless, these samples could be poled up to 3 kV/mm and magnetoelectric coefficients measured. Note the increase of the longitudinal *α*_33_ with poling at increasing electric fields (Figure 15a), due to the higher *d*_33_ values achieved. Note also the difference between the longitudinal and transverse responses (Figure 15b) after poling at 3 kV/mm (*d*_33_ of 5 pC/N).

The magnetoelectric response of two-phase composites depends on a variety of different issues, such as the inherent properties of the constituent phases (permittivity, conductivity or the piezo-coefficients) or the quality of interfaces between them, but also on the measuring mode used, that is, the orientation of the magnetic field with respect to the polarization direction [44]. For particulate 0–3 composites, a larger signal is typically achieved in longitudinal mode (*α*_33_), in which magnetic fields are oriented parallel to the poling direction and the output voltage. 

**Figure 16:**
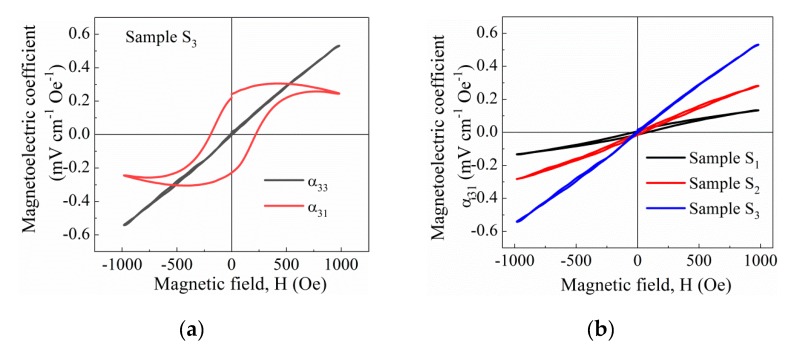
Room temperature magnetoelectric coefficients as a function of DC magnetic field of BNT–BT_0.08_/CoFe_2_O_4_ core–shell ceramics: (**a**) for sample with 1:1.5 molar ratio (sample *S*_3_) measured in both longitudinal (*α*_33_) and transverse (*α*_31_) modes after poling at 2 kV/mm, and (**b**) for ceramics with 1:0.5, 1:1 and 1:1.5 molar ratios (sample *S*_1_, sample *S*_2_ and sample *S*_3_).

Figure 16a shows the magnetoelectric voltage coefficients versus DC magnetic field at room temperature for the BNT–BT_0.08_/CoFe_2_O_4_ core–shell ceramic with highest molar ratio (sample *S*_3_), measured in both longitudinal (*α*_33_) and transverse (*α*_31_) modes. This sample S_3_ could only be poled up to 2 kV/mm and a Berlincourt *d*_33_ coefficient of 6 pC/N resulted. Poling at higher fields resulted in very large leakage currents, due to a higher conductivity about one order of magnitude greater in this sample as compared to sample *S*_1_ (see also Figure 11d). Note again the difference between longitudinal and transverse responses, which is highly linear in the former and quite hysteretic for the latter, but also note that higher d_33_ and magnetoelectric responses achieved for sample *S*_3_ despite the lower poling field applied.. It should further be noted that the ME response measured is truly as demonstrated by their relationship with the polarization direction of the piezoelectric phase. In particular, if the sample is placed in the direction of polarization and in the opposite direction to the bias magnetic field, one obtains negative sign voltage. It must be stressed that we are considering the voltage response to a linear AC magnetic field, which strongly depends on the bias magnetization field; this type of curve in composites rules out the Faraday induction, which is indeed taken into account in our measurements. The linear and non-hysteretic response found in these composites could be very advantageous for applications in sensors and oscillators with low noise and high sensibility.

This difference between longitudinal and transverse responses is related to the shape magnetic anisotropy of the disc samples [45]. Indeed, in magnetoelectric composites, these curves are tightly related to the effective piezomagnetic coefficient, which can be eventually extracted from the magnetization hysteresis curves [44]. A peak value should be obtained at the position of maximum piezomagnetic coefficient, that is, the bias field for maximum response in CoFe_2_O_4_ is typically above 1 kOe [46]. Note, finally, the higher longitudinal *α*_33_ achieved with increasing the content of magnetic phase in these core-shell composites (Figure 16b). This may likely be due to the greater *d*_33_ achieved at equal poling fields (that is, samples *S*_1_, *S*_2_ and *S*_3_ results in *d*_33_ of 2, 4 and 6 pC/N, respectively, after poling at 2 kV/mm). 

## 4. Conclusions

Multiferroic BNT–BT_0.08_/CoFe_2_O_4_ core–shell composites with uniform–nanosized grains were successfully fabricated using the sol–gel technique. The XRD analyses indicated the presence of two different crystallographic phases, BNT–BT_0.08_ and CoFe_2_O_4_. Additionally, from Rietveld analysis we showed that the values of the average crystallite size for CoFe_2_O_4_ phases decrease with increasing CoFe_2_O_4_ amount, whilst the tetragonality (*c*/*a* ratio) of the core shell samples decreases as the content of CoFe2O4 increases. As expected, the XPS results showed an increase in composition for Fe^3+^ and Co^2+^ ions with no modification of valence, thus confirming the X-Ray results. The TEM images proved the presence of core–shell microstructure of the BNT–BT_0.08_/CoFe_2_O_4_ nanocomposites.

Furthermore, the CoFe_2_O_4_ content has a significant influence on dielectric and magnetic properties, which consequently influences the magnetoelectric measurements. The magnetic properties of the BNT–BT_0.08_/CoFe_2_O_4_ revealed a close connection with the local structure of CoFe_2_O_4_. The remnant magnetization and saturation magnetization of the BNT–BT_0.08_/CoFe_2_O_4_ powders and ceramics increase with CoFe_2_O_4_ content. The coercivity increases with CoFe_2_O_4_ content except for the third sample, which has a higher CoFe_2_O_4_ contribution. This behavior could be related to a decrease of the stress anisotropy or to a decrease of the density of structural defects. The conductivity of the composites increases with increasing concentration of CoFe_2_O_4_. The piezoelectric and the magnetoelectric coefficients, measured in the longitudinal mode, increase and also with increase of the content of the magnetic phase. The maximum ME coefficient of the composite reaches up to about 0.6 mV/cm Oe.

Last, but not least, high attention should be focused on increasing the poling efficiency in this type of inverse core-shell magnetoelectric composites (with magnetic phase at the shell), in order to enhance the degree of polarization achieved, and thus the magnetoelectric voltage responses.

## Figures and Tables

**Table 1:** Rietveld refinement results for the BNT–BT_0.08_/CoFe_2_O_4_ core–shell powders.

Powders	*S*_1_: 1:0.5		*S*_2_: 1:1		*S*_3_: 1:1.5	
	BNT–BT_0.08_	CoFe_2_O_4_	BNT–BT_0.08_	CoFe_2_O_4_	BNT–BT_0.08_	CoFe_2_O_4_
a [Å]	5.5192	8.3774	5.5231	8.3872	5.5260	8.3777
b [Å]	5.5192	8.3774	5.5231	8.3872	5.5260	8.3777
c [Å]	3.9126	8.3774	3.9137	8.3872	3.9064	8.3777
c/a	0.7089		0.7086			0.7069
Volume [Å^3^]	119.1825	587.9251	119.3868	588.3788	119.2883	587.9922
*R*_exp_	5.8666		4.6676		4.2794	
*R*_p_	3.3093		2.6739		2.5936	
*R*_wp_	4.2908		3.4512		3.3441	
*χ* ^2^	0.5349		0.5467		0.6107	
Average crystallite size, D [nm]	28.32	26.76	31.64	25.26	29.04	22.16

**Table 2:** Dielectric properties measured at room temperature for the BNT–BT_0.08_/CoFe_2_O_4_ core–shell ceramics.

Sample	Permittivity *ε_r_*, at 1 kHz	Tan *δ*, at 1kHz	Conductivity *σ_dc_*, (*S*/cm)
*S*_1_, ceramic	515	0.632	1.66 × 10^−9^
*S*_2_, ceramic	468	1.086	6.27 × 10^−9^
*S*_3_, ceramic	318	1.750	1.36 × 10^−8^

**Table 3:** Hyperfine parameters, relative areas, inversion degree and formula units obtained from the Mössbauer spectra. The errors on the last digit are found in brackets.

Sample Code	Component	IS (mm/s)	QS (mm/s)	*B*_hf_ (*T*)	*R*_A_ (%)	*γ*	Formula Unit
*S*_1_	T	0.33(1)	−0.01(2)	50.1(1)	34(2)	0.708	(Co_0.292_Fe_0.708_)[Co_0.708_Fe_1.292_]O_4_
	O	0.271(4)	−0.014(8)	47.77(4)	66(2)		
*S*_2_	T	0.331(5)	−0.006(9)	50.1(4)	38(1)	0.789	(Co_0.211_Fe_0.789_)[Co_0.789_Fe_1.211_]O_4_
	O	0.271(2)	0.0	47.80(2)	62(1)		
*S*_3_	T	0.341(6)	0.0	50.48(5)	29(1)	0.606	(Co_0.394_Fe_0.606_)[Co_0.606_Fe_1.394_]O_4_
	O	0.275(3)	0.007(5)	47.78(2)	71(1)		

**Table 4:** Magnetic properties, measured at room temperature for the BNT–BT_0.08_/CoFe_2_O_4_ core–shell powders and ceramics.

Sample	CoFe_2_O_4_ Mass (out of 1)	*M_S_* (emu/g)	*M_R_* (emu/g)	*H_C_* (Oe)
*S*_1_, powder	0.35	24.7	10.5	1016
*S*_2_, powder	0.52	35.8	14.7	1199
*S*_3_, powder	0.62	47.3	20	1083
CoFe_2_O_4_ powder [32]	1	82.8	52	814
CoFe_2_O_4_ core – BaTiO_3_ shell powder composites [8]	0.6	33.9	5.98	399
CoFe_2_O_4_ core – BNT–BT_0.08_ shell composites [41]	0.975	73	61	60
CoFe_2_O_4_ – BNT–BT_0.65_ particulate composites [10]	0.1	2.5	0.5	3972
	0.2	7.5	1.5	4500
	0.3	13	2.5	4676
	0.4	17	3	6000
*S*_1_, ceramic	0.35	21.1	3.36	209
*S*_2_, ceramic	0.52	34.9	5.92	223
*S*_3_, ceramic	0.62	47.2	7.15	187
